# A System of Operative Surgery

**Published:** 1909-12

**Authors:** 


**A System of Operative Surgery.** Edited by F. F. BURGHARD, M.S., F.R.C.S. Vol. II. Pp. xxx, 720. London: Oxford Medical Publications. 1909.

The compilers of this *System* have been very fortunate in their choice of the specialists to whom the various articles have been entrusted. In most cases the authors have confined themselves to describing the methods of their own practice, and this gives great weight to their remarks.

Mr. Stiles writes on tuberculous disease of bones and joints, and he describes tuberculous disease of the diaphyses much more thoroughly than is done elsewhere. The wide removal of the shaft of the affected bone is advised in treatment in preference to mere scraping operations. Mr. Edmund Owen's account of hare lip and cleft palate does not give any definite discussion of the relative merits of the Langenbeck and Davies Colley methods. Brophy's method of approximating the jaws in early infancy is recommended.

Mr. Fagge's article on operations on the jaws is chiefly notable for the useful and judicious references to literature and statistics. Mr. Butlin, whose unique experience of tongue surgery enables him to speak with unrivalled authority, contributes a most valuable article on this subject, which contains a careful summary of all his own cases of excision of cancer of the tongue, numbering 200. The routine use of preliminary laryngotomy in all cases of tongue excision is important. The muscles of the tongue are to be removed when possible right down to the hyoid bone. In operating on cancer of the back of the tongue, the hyoid bone is usually divided, but the tip of the tongue is preserved. These latter operations, whilst giving excellent immediate results, have so far had no ultimate success. In the description of the removal of cancer of the tonsil no account is given of the operation through an external incision, with division of the jaw.

Mr. Fagge's article on the œsophagus is clear and up to date, including an account of trans-mediastinal operations, but mentioning only the negative differential pressure methods, and not the positive.

Mr. Moynihan's article on gastric surgery is a model of clearness and precision, and his minute description of details makes all that is said of great practical value.

Mr. Makin's article on intestinal surgery contains a very great wealth of information, collected both from personal experience and from current literature. In dealing with the colon especially, much recent work is brought together for the first time in text-book form. The relation of the lymph vessels and glands to the different parts of the large intestine, and the operations for their removal, are carefully discussed.

Mr. Barker has undertaken the section on hernia. In this the author has had the difficult task of selecting what is best worth description out of the vast mass of operative methods. As regards inguinal hernia, we think that undue prominence is given to Kocher's and Halsted's methods, whilst such points as the fixation of the stump of the sac by sewing inside the abdominal parietes, or the attachment of the internal oblique to Poupart's ligament in front of the cord, are not mentioned.

The volume concludes with an article on the rectum and anus by Mr. Swinford Edwards. This is very clear and complete, especially in the description of the lateral sacral methods of excision. Perhaps a little more space might have been allotted to the section on removal of the rectum by the combined routes.

As a whole, the volume is characterised by two most excellent points, both of which will ensure its appreciation by students and practitioners. The one is that all the authors speak chiefly of methods in which they themselves have had large experience; the other is the uniform excellence of the illustrations, which reflects the highest credit on the publishers, authors, and artists alike.[1]

[1] The publishers of this *System* have drawn our attention to the review in our last number on the first volume of the *System* as exceeding the limits of reasonable criticism. Our reviewer finds that he was in error when he quoted the author of the article on "Displaced Knee Cartilages" as having recommended immediate massage and movement for the condition. The word "immediate" was not used in the text, and the author's meaning was misunderstood; we therefore unreservedly withdraw this statement, and at the same time desire to express our regret that the remarks made on certain illustrations in another part of the book were unduly severe.

